# Hypertension and Dyslipidemia: the Two Partners in Endothelium-Related Crime

**DOI:** 10.1007/s11883-023-01132-z

**Published:** 2023-08-18

**Authors:** Edyta Dąbrowska, Krzysztof Narkiewicz

**Affiliations:** 1 Center of Translational Medicine, Medical University of Gdańsk, Dębinki 7, 80-952 Gdańsk, Poland; 2 Department of Hypertension and Diabetology, Medical University of Gdańsk, Smoluchowskiego 17, 80-214, Gdańsk, Poland

**Keywords:** Hypertension, Dyslipidemia, Endothelial dysfunction, Metabolomics, Artificial intelligence

## Abstract

**Purpose of Review:**

The goal of this article is to characterize the endothelium’s role in the development of hypertension and dyslipidemia and to point out promising therapeutic directions.

**Recent Findings:**

Dyslipidemia may facilitate the development of hypertension, whereas the collaboration of these two silent killers potentiates the risk of atherosclerosis. The common pathophysiological denominator for hypertension and dyslipidemia is endothelial cell dysfunction, which manifests as dysregulation of homeostasis, redox balance, vascular tone, inflammation, and thrombosis. Treatment focused on mediators acting in these processes might be groundbreaking. Metabolomic research on hypertension and dyslipidemia has revealed new therapeutic targets. State-of-the-art solutions integrating interview, clinical examination, innovative imaging, and omics profiles along with artificial intelligence have been already shown to improve patients’ risk stratification and treatment.

**Summary:**

Pathomechanisms underlying hypertension and dyslipidemia take place in the endothelium. Novel approaches involving endothelial biomarkers and bioinformatics advances could open new perspectives in patient management.

## Introduction

Hypertension and dyslipidemia constitute the major risk factors for cardiovascular (CV) diseases [1]. Despite extensive research efforts, the availability of diagnostic tools, and effective treatment, they remain the leading causes of CV mortality and disability-adjusted life years worldwide [2•, 3, ]. The coexistence of hypertension and dyslipidemia potentiates their deleterious impact on the CV system compared to the sum of their individual effects [1, 4–6]. Indeed, the INTERHEART study revealed that a single risk factor multiplies the total risk from twofold to threefold, whereas the coincidence of hypertension, dyslipidemia, diabetes mellitus, and smoking leads to a more than 20-fold increase in the total risk [6]. Interestingly, it has been shown that therapeutic approaches focused on both hypertension and dyslipidemia may result in significantly greater CV risk reduction [5, 7, 8]. This bilateral synergistic effect indicates common pathomechanisms underlying both hypertension and dyslipidemia and reveals a significant role for endothelial dysfunction. In hypertension, endothelial cell dysfunction accelerates the development of the deleterious consequences of dyslipidemia [9], and conversely, the remodeling of the vascular wall present in dyslipidemia complicates the course of hypertension. Therefore, in this review article, we trace recent discoveries in endothelial alterations participating in dyslipidemia and hypertension and highlight their role in initiating atherosclerotic disease. Furthermore, we point out new directions for patient management involving the use of artificial intelligence to integrate clinical data with the latest omics results [10•] to identify high-risk patients and personalize treatment plan.

## Hypertension and Endothelial Dysfunction

A proper understanding of hypertension requires addressing the endothelium’s pivotal role in blood flow regulation. Endothelial cells produce many vasoactive substances, among which nitric oxide (NO) has the greatest vasodilating potential [11–13], leading to vascular smooth muscle relaxation via activation of guanylate cyclase and generation of intracellular cyclic guanosine monophosphate [11, 14]. Endothelial NO synthase (eNOS) produces NO from L-arginine and is stimulated by (1) receptor-dependent agonists, such as acetylcholine and bradykinin, (2) non-receptor-dependent agonists, such as calcium ionophores, and (3) blood flow [15–17]. Vasodilating agents (i.e., prostacyclin, calcitonin gene-related peptide, adrenomedullin, and substance P) produced by a variety of cell types also lead to NO secretion in endothelial cells [11, 18, 19]. In contrast, the endothelium is also responsible for the release of vasoconstrictors, such as endothelin 1, locally generated angiotensin II, thromboxane A2, and prostaglandin A2 [11, 20]. Endothelin 1 links to the ET-A receptor and, apart from smooth muscle constriction, promotes the activity of other vasoconstrictors. Growth factors linked to vasoconstrictive substances stimulate matrix modification in the vascular wall and its remodeling [11].

The role of oxidative stress in endothelial dysfunction is crucial. Angiotensin II, a key component of this process, stimulates NADPH/NADH oxidase in the endothelium, smooth muscle cells, and adventitia to produce reactive oxygen species (ROS) [21–23]. NADPH/NADH oxidase can additionally enhance the production of superoxide (O_2_^−^) from mitochondria and xanthine oxidase, which reacts with NO, resulting in peroxynitrite. eNOS uncoupling occurs through the following mechanisms: (1) deficiency of the cofactor tetrahydrobiopterin through its oxidation by peroxynitrite or (2) deficiency of the substrate L-arginine attributed to increased arginase expression observed in hypertension [24–27], and (3) S-glutathionylation observed in angiotensin II-induced hypertension [28]. Importantly, uncoupled eNOS produces superoxide (O_2_^−^), driving the vicious circle of oxidative stress. Finally, eNOS uncoupling decreases NO production and promotes vascular inflammation and remodeling [24]. Surprisingly, eNOS overexpression leads to its uncoupling due to a relative deficiency of the cofactor tetrahydrobiopterin [24, 29, 30].

Endothelial NO production is heavily dependent on hemodynamics. Laminar blood flow activates eNOS, while disturbed flow or oscillatory shear stress decreases eNOS expression, contributing NADPH/NADH oxidase and xanthine oxidase to superoxide production [24, 31, 32] and exposing vessel walls to oxidative stress. It is noteworthy that the pattern of hemodynamic stimulation (undisturbed vs. disturbed flow) is associated with the activation of transcriptional factors participating in the expression of genes responsible for the functional endothelial phenotype [15]. Laminar flow leads to upregulation of transcription factors such as Kruppel-like factor (KLF)-2, KLF-4, and nuclear factor erythroid 2-related factor 2 (Nrf-2), resulting in an atheroprotective phenotype [15, 33]. On the contrary, oscillatory flow leads to upregulation of the pleiotropic transcription factor NF-κB, which orchestrates an atheroprone phenotype [15, 33]. Interestingly, KLF-2 and Nrf-2 are responsible for 70% of the atheroprotective endothelial transcriptome, pointing to a new area for targeted therapeutic approaches [15, 34].

## Dyslipidemia and Endothelial Dysfunction

The link between dyslipidemia and endothelial dysfunction has been shown in many studies [35–37]. Low-density lipoprotein (LDL) is responsible for endothelial ROS production. Lipid peroxidation occurs through nonenzymatic processes (by ROS derived from NADPH/NADH oxidase or uncoupled eNOS) or enzymatic processes (performed by lipoxygenases, myeloperoxidase, and cyclooxygenases). Lipid peroxidation products generate oxidation-specific epitopes (OSEs) on the surface of oxidized LDL (oxLDL) molecules [24, 38, 39]. An OSE is recognized by receptors (i.e., scavenger receptors, toll-like receptors, mediators of the complement system, or IgM antibodies). Genetic research has shown that deletion of lipoxygenases decreases LDL oxidation and the process of atherosclerosis in mice [24, 40], and OSE-specific natural IgM antibodies inhibit the uptake of LDL by macrophages and prevent foam cell formation in mice [24, 41, 42]. Endothelial cells and macrophages—as major sensors of OSE—uptake oxLDL, which has a wide array of proatherogenic properties. Additionally, scavenger receptors are not downregulated by an LDL increase; therefore, LDL can easily accumulate and generate foam cells—the first step of atherosclerosis. Virchow, based on autopsy studies, emphasized that lipid accumulation occurs at the sites of early endothelial lesion formation [15, 43]. Moreover, several studies have shown that disturbed patterns of flow observed in arterial curvatures, bifurcations, and side branches favor the development of atherosclerosis. In such regions, endothelial cells display cuboidal morphology, higher cell turnover, and an impaired endothelial barrier function, leading to migration of LDL and inflammatory mediators. In contrast, regions exposed to laminar flow exhibit ellipsoidal cell morphology, coaxial alignment, and glycocalyx, providing protection from lipoprotein extravasation [44–47].

## Interplay Between Hypertension and Dyslipidemia Accelerates Atherosclerosis Development

The common vascular result of hypertension and dyslipidemia is endothelial dysfunction that may lead to atherosclerosis. These two risk factors, through overlapping pathomechanisms occurring at the level of the endothelium, lead to striking atherosclerosis progression. Previous studies have reported results supporting the interplay between hypertension and dyslipidemia in CV disease aggravation [4, 48–51]. In Watanabe heritable hyperlipidemic rabbits, induced hypertension significantly accelerated the development of atherosclerosis and plaque destabilization, leading to cardiac death [9]. In the Multiple Risk Factors Interventional Trial, it was demonstrated that even mild to moderate levels of both hypertension and dyslipidemia had a multiplicative adverse impact on the risk of coronary heart disease [4, 7, 49]. A study of young patients with a family history of hypertension and borderline blood pressure values [4, 52] showed a significant increase in the 10-year relative risk of developing hypertension in the patients with baseline cholesterol levels above the cut-off value of 200 mg/dl. Moreover, strong evidence of the interplay between dyslipidemia and hypertension comes from studies demonstrating that the use of statins in hypertensive patients can favorably affect individual risk profiles by interacting with blood pressure control [4, 53, 54]. Interestingly, it has been shown that therapeutic approaches focused on both hypertension and dyslipidemia may result in significantly greater CV risk reduction [5, 7, 8].

In hypertension, dyslipidemia may aggravate the development of atherosclerosis through the following mechanisms. First, chronic oscillatory shear stress—driving oxidative stress, redox imbalance, and upregulation of lipid oxidation enzymes—leads to LDL oxidation and internalization [55, 56]. Second, elevated blood pressure enhances angiotensin II binding to the angiotensin type 1 (AT1) receptor, which results in augmented lipid uptake in the vessel wall [55, 57].

In dyslipidemia, through stimulation of LDL to ROS production and through eNOS uncoupling, NO bioavailability is diminished, which contributes to vasoconstriction. Second, hypercholesterolemia enhances arginase activity—an enzyme competing with eNOS for L-arginine, which results in eNOS uncoupling with its further consequences. Third, dyslipidemia leads to upregulation of the AT1 receptor, enhancing the vasoconstrictive effect of angiotensin II [24, 58]. Fourth, it has been proven that dyslipidemia increases arterial stiffness, predisposing to the development of hypertension [59, 60]. Finally, dyslipidemia by reducing baroreflex sensitivity impairs the negative feedback loop and dysregulates blood pressure control [61, 62].

The magnitude of the reactions taking place at the endothelium level directs our considerations toward microcirculation—the part of a vascular tree having an area advantage over other parts. Microvessels constitute approximately 99% of all vessels in the human body, and their total surface area is estimated at 500 to 700 m^2^ [63]. Therefore, assessment of the endothelium in the microcirculation area-such a hemodynamically significant modulator with an impressive surface —may provide insights into CV status.

A decline in endothelial-vasodilating properties and inflammation process generating a neointima in response to the deleterious effect of hypertension and dyslipidemia lead to vascular remodeling. Although both diseases are indirectly associated with arterial occlusion, the distribution pattern of these diseases in the arterial tree is different [64]. In hypertension, the lumen narrowing process is observed in small vessels and microvascular bed, while in large vessels, intima media thickening and vessel enlargement occur. In atherosclerosis, obstructive lesions are localized in medium and large vessels. Cross talk between micro- and macrocirculation aggravates and accelerates these unfavorable alterations in the CV system. Small arteries are the major determinant of total peripheral resistance. Microvessel remodeling, manifested as a reduced lumen diameter and an increased wall-to-lumen ratio, leads to an increase in total peripheral resistance and blood pressure values [65–68]. Additionally, stiff components are loaded in the arterial wall, subsequently enhancing large artery stiffness, which is related to a decreased ability to accommodate the volume of blood ejected from the left ventricle [67–69]. Arterial stiffness leads to an increase in systolic and pulse pressures and a shift of reflection sides toward microvessels. Then, pressure pulsatility penetrates the microcirculation, resulting in further vessel remodeling and organ damage [70]. This vicious circle of successive hypertension processes might be dizzyingly accelerated by accompanying diseases such as dyslipidemia due to the common pathophysiological background taking place in endothelial dysfunction.

## Metabolomic Profiles of Patients with Hypertension and Dyslipidemia

Metabolomic studies hold promise for providing insights into current knowledge on the pathophysiology, progression, and prognosis of hypertension or dyslipidemia. Furthermore, metabolomic analyses will open up new perspectives for personalized treatment. However, investigations focused on developing and validating metabolomic models in hypertension or dyslipidemia are still very scarce.

A few studies have addressed the plasma metabolomic profile of hypertension in relation to lipids. The prospective investigation by Lin et al., including 504 men and women with a follow-up period of over 5 years aimed to identify associations between circulating metabolites and longitudinal blood pressure progression [71, 72]. Metabolomic pathways, including glycerolipids, ceramide, triacylglycerol, oleic acid, and cholesteryl ester, were associated with longitudinal changes in diastolic blood pressure [72]. In Kulkarni et al.’s longitudinal study, the association between the plasma lipidomic profile and hypertension was evaluated [73]. The results demonstrated that diacylglycerols were significantly associated with systolic, diastolic, and mean blood pressure, as well as the risk of incident hypertension during 7140.17 person-years of follow-up [73]. Furthermore, Dietrich’s study of serum metabolites revealed that phosphatidylcholines had predictive value for hypertension development during the 10-year follow-up period [74]. In the research of Chaofu Ke, the common metabolic pattern of upregulated diglycerides and lysophosphatidylcholine for both hypertension and dyslipidemia was found [1].

Interestingly, all the above investigations highlighted the significant role of lipid metabolites in hypertension development. Lipids are divided into the following eight categories: sphingolipids (i.e., ceramide), glycerolipids (triacylglycerol), fatty acyls (i.e., oleic acid and cholesteryl ester), phospholipids, sterol lipids, prenol lipids, saccharolipids, and polyketides [72, 75, 76]. Some were revealed in the aforementioned studies to be significant in hypertension development, contributing to endothelial dysfunction by inhibiting endothelium-derived vasodilating mediators or stimulating endothelium-dependent vasoconstrictors [72]. For instance, ceramides, by inhibiting the eNOS-serine/threonine protein kinases-heat shock protein 90 signaling complex and enhancing thromboxane A2, lead to endothelial disability and vasoconstriction [77, 78]. These data elucidate the role of lipidomic pathways in blood pressure regulation and the etiopathogenesis of hypertension. Since the metabolic pathways of hypertension and dyslipidemia partially overlap, the frequent coincidence of these two diseases seems obvious. Therefore, therapy focused on both hypertension and dyslipidemia may multiply its beneficial effects and result in greater CV disease risk reduction.

## Therapeutic Approaches and the Potential of Artificial Intelligence

**Fig. 1:**
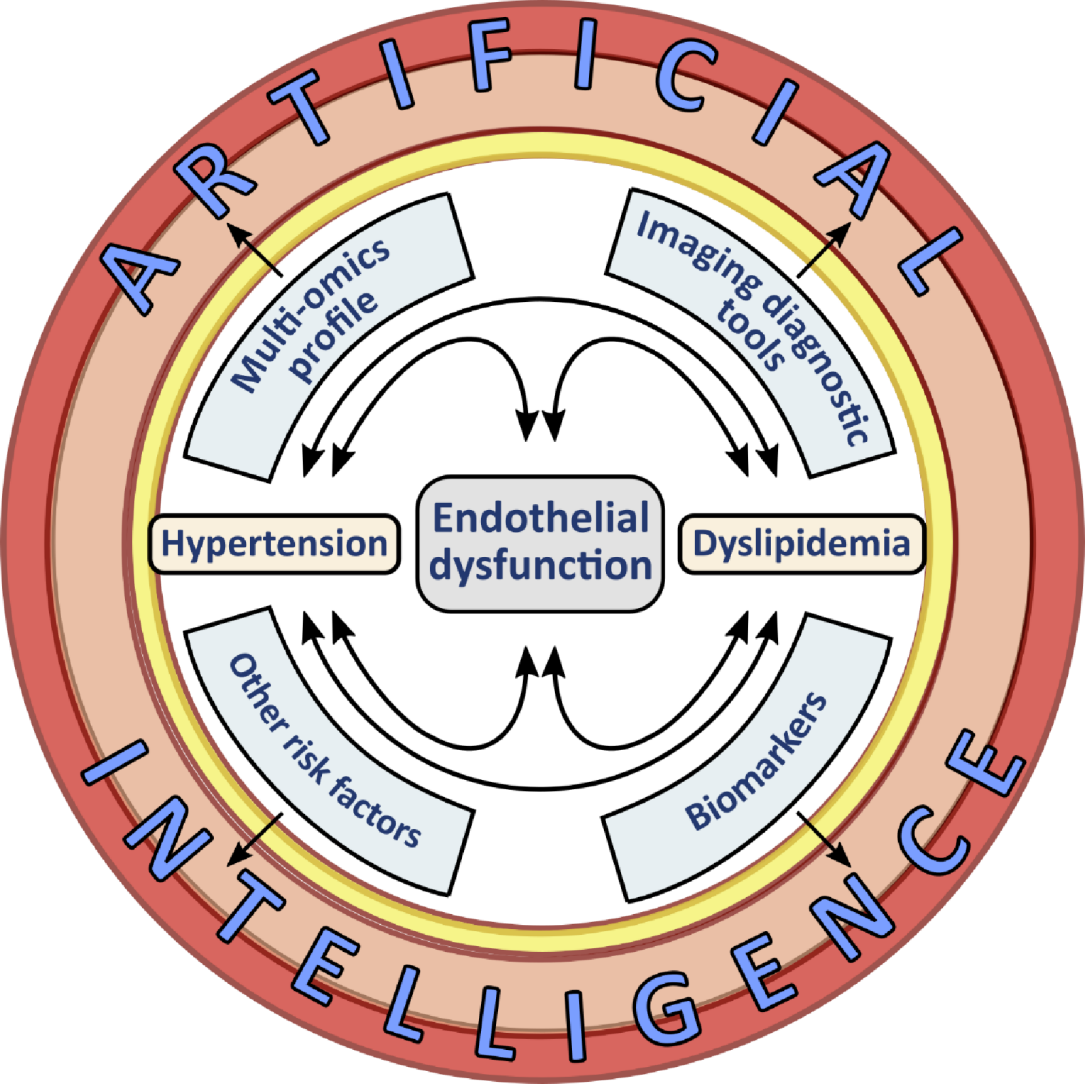
Data integration by novel bioinformatics tools

A combination of genetic predisposition, the metabolomic profile signature, and early phenotypic features, including endothelial dysfunction hallmarks, may lay the foundation for early identification of presymptomatic patients and initiation of treatment management plans. Due to the growing global prevalence of hypertension and dyslipidemia [71, 79, 80], new risk stratification scores for patients’ personalized treatment strategy are needed. However, in the face of an abundance of data obtained in omics research, traditional regression analyses start to be insufficient, mainly because the predictor’s number importantly exceeds the patient’s number [81, 82]. For these purposes, novel, more sophisticated approaches might be applied [81]. Artificial intelligence has opened a new chapter in biostatistics [81]. Machine learning—a rapidly improving branch of artificial intelligence—allows the integration of clinical data and the genetic background with recent metabolomics results (Fig. 1), which might be translated into screening, diagnostic, and treatment plans [2•]. It opens up a promising perspective for improving current risk stratification and implementing multidimensional personalized medicine.

## Conclusions

The growing prevalence of hypertension and dyslipidemia calls for novel solutions. Assessment of endothelial biomarkers is of great importance, and it constitutes a high-potential tool in the future diagnosis of CV diseases. The metabolomic signature brings new insights into early alterations preceding the development of hypertension [74] and dyslipidemia, directing toward new therapeutic approaches. However, the scarce number of prospective studies indicates the need for further research. Due to innovative bioinformatics tools, a huge amount of data obtained from different diagnostic resources might be analyzed for more sensitive identification of high-risk patients [2•, 81]. Personalized medicine opens prospects for reducing the global burden of hypertension and dyslipidemia.
